# Transcriptomic analysis provides insight into defensive strategies in response to continuous cropping in strawberry (*Fragaria* × *ananassa* Duch.) plants

**DOI:** 10.1186/s12870-022-03857-6

**Published:** 2022-10-07

**Authors:** Peng Chen, He-qin Li, Xing-yue Li, Xian-hong Zhou, Xiu-xia Zhang, An-sheng Zhang, Qi-zhi Liu

**Affiliations:** 1 Key Laboratory of Natural Enemies Insects, Ministry of Agriculture and Rural Affairs, Shandong Provincial Engineering Technology Research Center on Biocontrol of Crop Diseases and Insect Pest, Institute of Plant Protection, Shandong Academy of Agricultural Sciences, 250100 Jinan, China; 2 Laboratory of Entomology and Nematology, College of Plant Protection, China Agricultural University, 100193 Beijing, China; 3 Shandong Provincial Key Laboratory of Dryland Technology, College of Agronomy, Qingdao Agricultural University, 266109 Qingdao, China; 4 Institute of Plant Protection, Sichuan Academy of Agricultural Science, 610066 Chengdu, China

**Keywords:** Continuous cropping, WRKY transcription factor, Peroxidase, Salicylic acid

## Abstract

**Background:**

Strawberries are an important economic fruit crop world-wide. In strawberry cultivation, continuous cropping (CC) can seriously threaten yield and quality. However, our understanding of the gene expression changes in response to CC and during subsequent defense processes is limited. In this study, we analyzed the impact of CC on the transcriptome of strawberry roots using RNA-Seq technology to elucidate the effect of CC and the subsequent molecular changes.

**Results:**

We found that CC significantly affects the growth of strawberry plants. The transcriptome analysis identified 136 differentially expressed genes (DEGs), including 49 up-regulated and 87 down-regulated DEGs. A Gene Ontology (GO) analysis indicated that the up-regulated DEGs were mainly assigned to defense-related GO terms, and most down-regulated DEGs were assigned to nutrient-related GO terms. Kyoto Encyclopedia of Genes and Genomes (KEGG) analysis revealed that the responsive DEGs were classified in a large number of important biological pathways, such as phenylalanine metabolism, starch and sucrose metabolism, phenylpropanoid biosynthesis, glutathione metabolism and plant-pathogen interaction. We also found that four WRKY transcription factors and three *peroxidase* genes involved in plant defense pathways were up-regulated in the roots of strawberry plants subjected to CC.

**Conclusion:**

Several unigenes involved in plant defense processes, such as *CNGCs*, *WRKY* transcription factors, *PR1*, and *peroxidase* genes with highly variable expression levels between non-CC and CC treatments may be involved in the regulation of CC in strawberry. These results indicate that strawberry roots reallocate development resources to defense mechanisms in response to CC. This study will further deepen our understanding of the fundamental regulatory mechanisms of strawberry resource reallocation in response to CC.

**Supplementary information:**

The online version contains supplementary material available at 10.1186/s12870-022-03857-6.

## Introduction

Strawberry (*Fragaria* × *ananassa* Duch., 2n = 8x = 56), which is famous for its delicious taste and attractive appearance, is the world’s most economically important fruit crop [1, 2]. Strawberry is a typical annual greenhouse plant, and it is sensitive to continuous cropping (CC) [1, 3]. In agricultural production, CC seriously impedes the sustainable development of strawberry farming, and substantial agricultural losses are caused by CC worldwide [4]. As the requirement to produce strawberries increases and arable land availability decreases, the challenges associated with CC represent a major obstacle requiring an urgent solution.

Most previous studies in this area have focused on identifying the factors that cause problems associated with CC [5, 6]. We previously reported that these problems are caused by complex factors in the soil, including changes in soil fertility, the accumulation of phenolic acids, and changes in bacterial, fungal, and nematode communities [3, 4, 7, 8]. The CC of strawberry leads to three phases of change in soil. Phase I includes significant alterations to soil physicochemical properties; during phase II, key changes to fungal nematode populations occur; and in phase III, the abundance of key bacteria changes significantly, while the accumulation of phenolic acids starts to inhibit crop growth significantly [9].

Plants respond to variable environments with a series of specific strategies controlled by a regulatory network [10]. Through this network, a series of physiological, molecular, and metabolic processes are activated to offer protection to the plant [11]. Plants also activate genes encoding stress response proteins such as transcription factors and other signaling molecules [12]. Transcriptomic analyses in model plants under controlled conditions have shown that the expression of hundreds of genes alters in response to different stresses. Yang et al. identified a set of 89 conserved and six novel miRNAs in *Rehmannia glutinosa* plants subjected to CC [13]. Meanwhile, Li et al. provided evidence of metabolic alterations in *Andrographis paniculata* after CC [14]. Moreover, Chen et al. found a possible recovery strategy in strawberry roots after the remediation of CC soil, including an overall decrease in nine defense-related *Hsp* genes [15]. However, the molecular basis of the response to CC in strawberry remains unclear.

Understanding the strategy implemented to recover from CC in strawberry is important, but it is also necessary to recognize the mechanisms of CC tolerance in this crop. For this purpose, we used RNA-Seq technology to analyze the differential expression of genes in the roots of strawberries subjected to CC and non-CC (NCC) and determine the key genes involved in the regulation of CC tolerance. This work will provide a valuable reference for unveiling the molecular mechanisms that underlie the direct response to CC in strawberry roots.

## Results

### Strawberry growth in response to continuous cropping

**Fig. 1:**
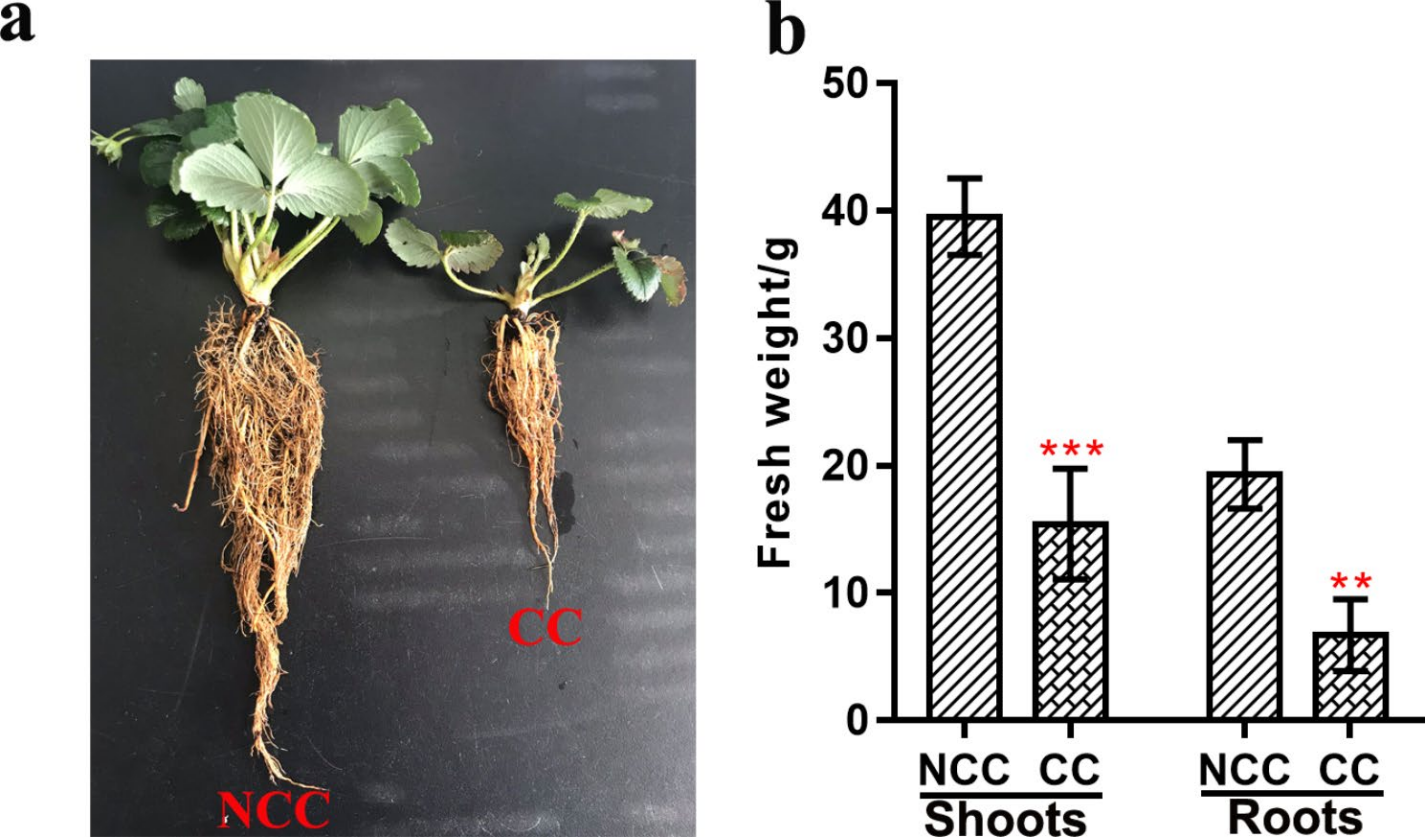
The effect of CC on growth in strawberry plants.(a) The growth status of strawberry plants. (b) The fresh weights of shoots and roots of the strawberry plants, ** *P* < 0.05; *** *P* < 0.01

Strawberry growth differed dramatically between NCC and CC treatments (Fig.1a). The fresh weights of the shoots and roots of the strawberry plants were significantly influenced by CC (Fig.1b). The fresh weights of the shoots and roots were very significantly (*P* < 0.01) reduced by CC, with decreases of 60.94% and 65.29%, respectively, in comparison with plants grown under the NCC treatment (Fig.1b).

### Illumina HiSeq mRNA sequencing

In this study, six libraries (NCC-1, NCC-2, NCC-3, CC-1, CC-2, and CC-3) were constructed for reference transcriptome sequencing and RNA-Seq analysis. Each library generated more than 7,482,488,270 base numbers and 25,068,546 clean reads. The percentage of high-quality (Q > 30) reads was > 91.54%, and 73.64–76.17% of the clean reads were mapped to the reference genome [2] (Table1).

**Table 1:** Summary of read mapping for RNA-seq

Samples	Base Number	GC Content	%≥Q30	Clean Reads	Mapped Reads	Mapped Ratio
NCC1	8,331,519,048	46.83%	92.93%	27,883,986	21,239,906	76.17%
NCC2	8,085,416,338	46.63%	92.36%	27,069,944	20,384,532	75.30%
NCC3	7,482,488,270	46.93%	93.09%	25,068,546	19,069,627	76.07%
CC1	7,675,408,282	46.49%	92.34%	25,742,493	19,008,544	73.84%
CC2	8,284,684,432	46.69%	92.22%	27,771,632	20,451,929	73.64%
CC3	8,931,237,260	47.41%	91.54%	29,900,828	22,380,673	74.85%

**Fig. 2:**
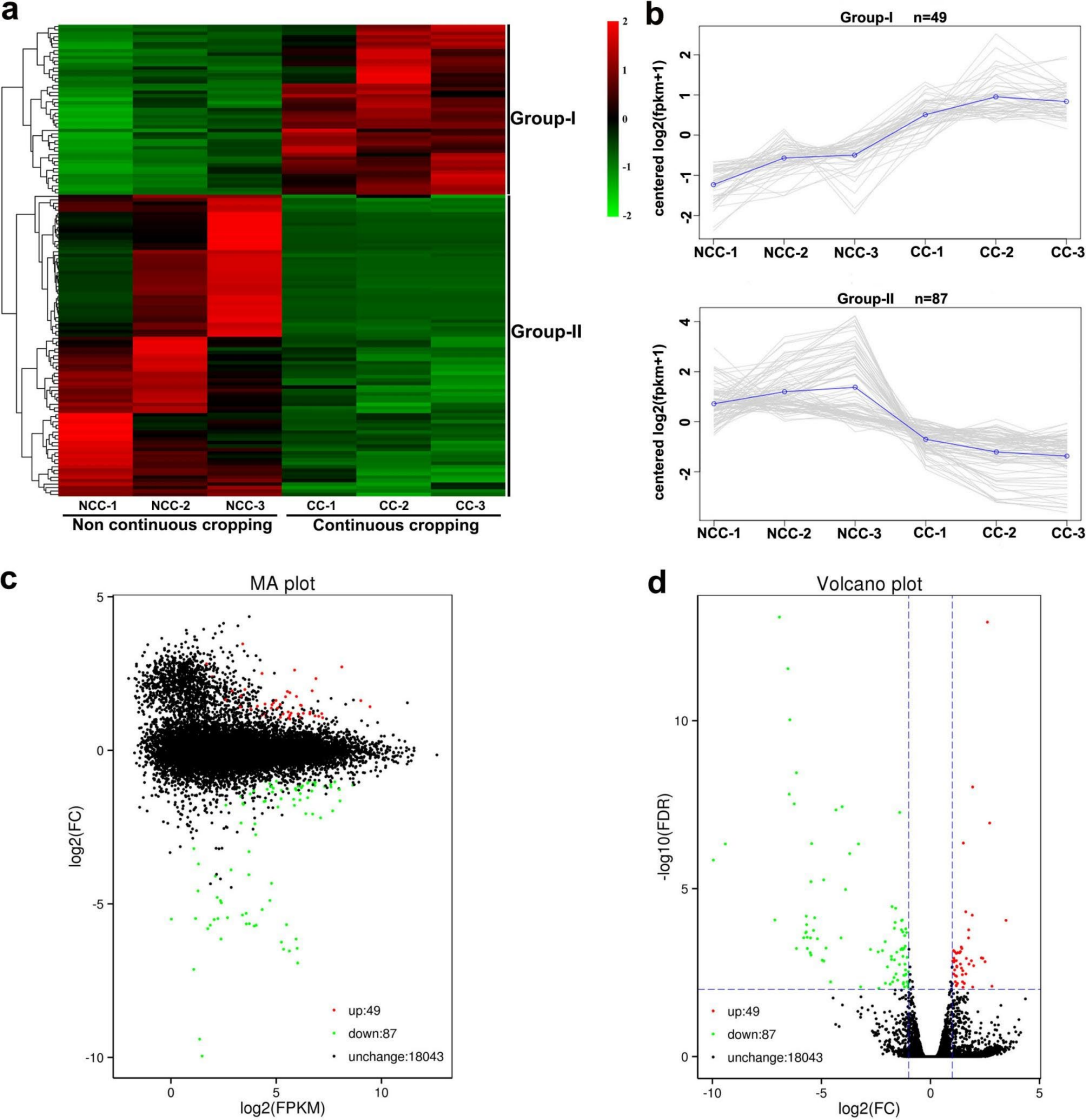
Expression analysis of the DEGs between NCC- and CC-treated strawberry roots. (a) H-cluster heatmap analysis. The columns represent different samples, and the rows represent different genes. The color change from green to red indicates low to high gene expression, respectively. (b) Gene co-expression trend analysis. The individual lines represent different genes.(c) MA plot. (d) Volcano plot. Each point in the MA plot and volcano plot represents a gene. The green points represent downregulated genes, the red points represent upregulated genes, and the black points represent unchanged genes

We performed a detailed comparative analysis to identify the global changes in the differentially expressed genes (DEGs) in two comparisons. A total of 136 DEGs were identified by the two comparisons, including 49 up-regulated and 87 down-regulated genes (Fig.2a-b). The hierarchical cluster analysis of all the DEGs indicated that all 136 could be classified into two subclusters (Fig.2a-b). The overall distribution of the gene expression abundances and differential fold changes between the two groups were visualized with an MA plot (Fig.2c). The differences in gene expression levels between the two groups are shown in a volcano plot (Fig.2d).

### Gene ontology enrichment analysis of DEGs

**Fig. 3:**
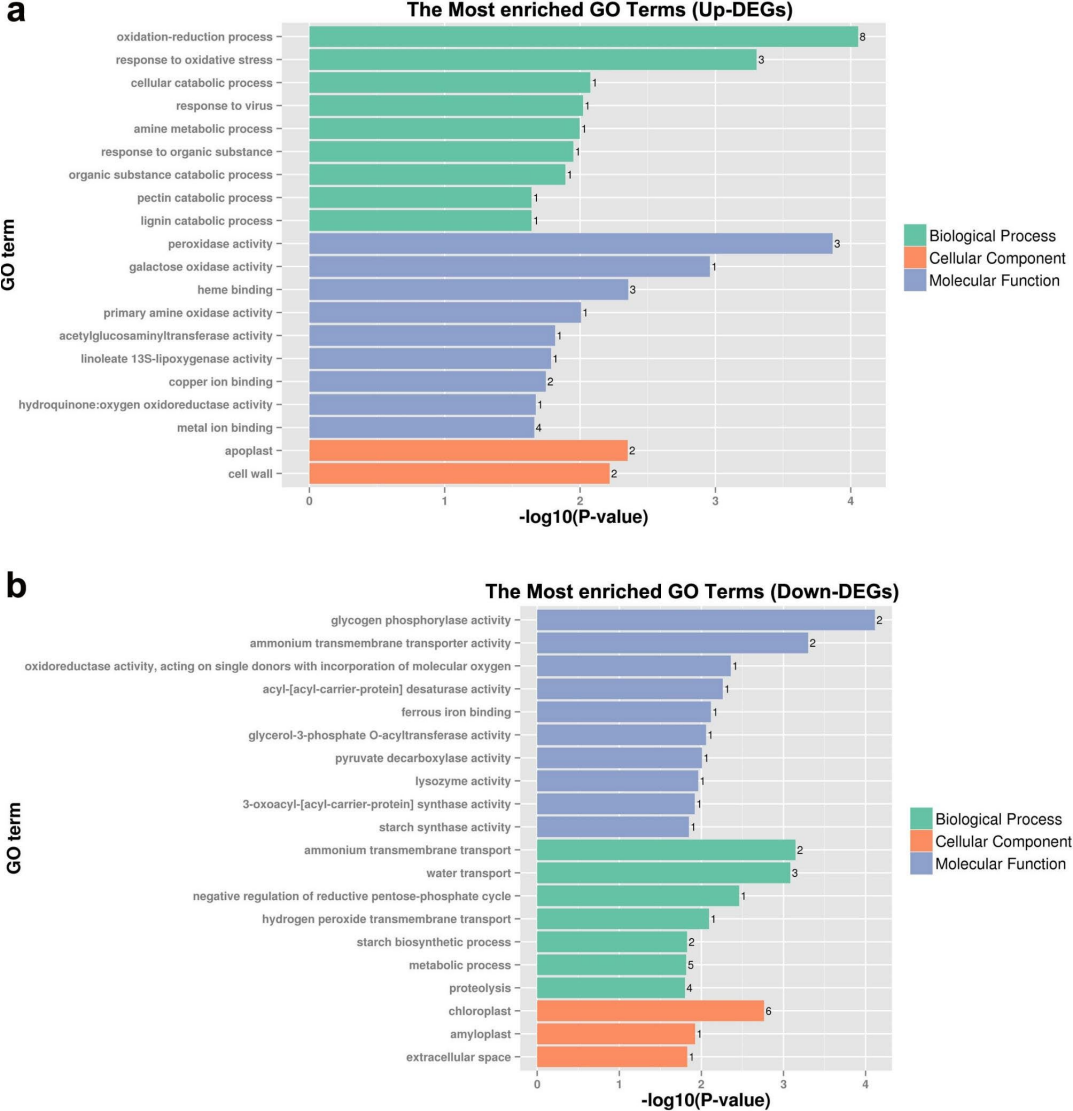
GO enrichment of DEGs. (a) GO enrichment of up-regulated DEGs (*P* < 0.05). (b) GO enrichment of down-regulated DEGs (*P* < 0.05). GO terms belonging to biological process, cellular component, and molecular function are shown in green, orange, and blue, respectively. The number of DEGs annotated to a GO term is marked at the top of each bar

In this study, gene ontology (GO) term enrichment analyses were used to classify the functions of the annotated DEGs. The up- and down-regulated DEGs were annotated with GO terms and assigned to biological process, cellular component, and molecular function (Fig.3). Many up-regulated DEGs were assigned to defense-related GO terms, which included: response to oxidative stress (GO:0006979), response to virus (GO:0009615), response to organic substance (GO:0010033), and lignin catabolic process (GO:0046274) under biological process; peroxidase activity (GO:0004601) under molecular function; and cell wall (GO:0005618) under cellular component (Fig.3a). Among the down-regulated DEGs, most were assigned to nutrient-related terms, including those associated with nutrient transport and synthesis. The nutrient transport-related terms included: ammonium transmembrane transport (GO:0072488), water transport (GO:0006833), and hydrogen peroxide transmembrane transport (GO:0080170) under biological process; and ammonium transmembrane transporter activity (GO:0008519) under molecular function (Fig.3b). The nutrient synthesis-related terms included: negative regulation of reductive pentose-phosphate cycle (GO:0080153) and starch biosynthetic process (GO:0019252) under biological process; glycogen phosphorylase activity (GO:0008184), pyruvate decarboxylase activity (GO:0004737), and starch synthase activity (GO:0009011) under molecular function; and chloroplast (GO:0009507) under cellular component (Fig.3b).

### KEGG pathway analysis of DEGs

**Fig. 4:**
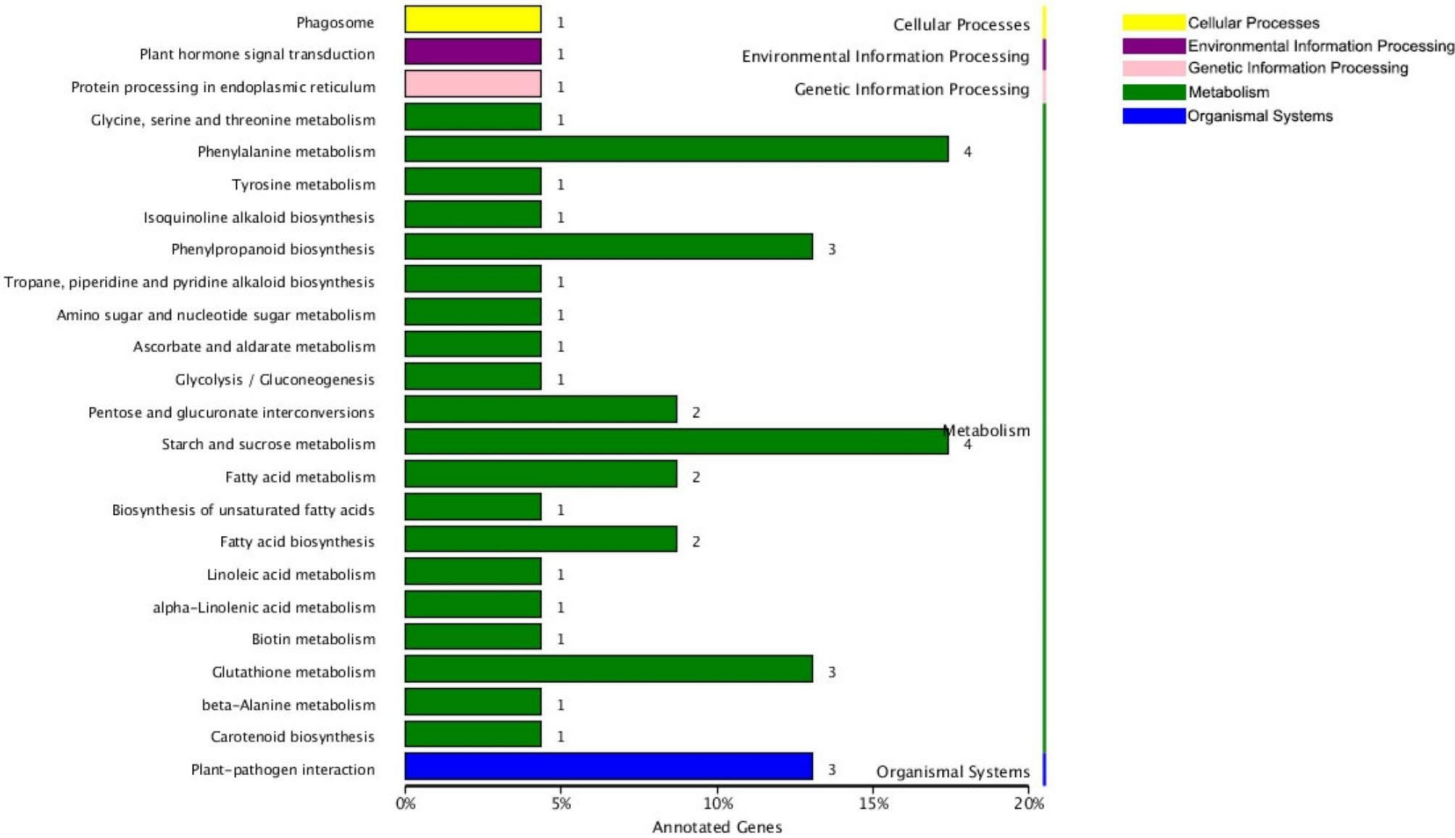
KEGG pathway enrichment analysis of DEGs. The number of DEGs annotated for each pathway is marked next to each bar. The different-colored bars represent different pathway categories. The cellular processes categories are indicated in yellow color; the environmental information processing categories are indicated in purple color; the genetic information processing categories are indicated in pink color; the metabolism categories are indicated in green color; and the organismal systems categories are indicated in blue color

To further reveal the functions of the DEGs in response to CC, KOBAS 2.0 was used to perform a KEGG enrichment analysis. Twenty-four pathways were found to be significantly enriched in five main categories (Fig.4). Most of the DEGs were assigned to metabolism, including four each to phenylalanine metabolism (ko00360) and starch and sucrose metabolism (ko00500) and three each to phenylpropanoid biosynthesis (ko00940) and glutathione metabolism (ko00480), respectively. Moreover, three DEGs were assigned to plant-pathogen interaction (ko04626) in organismal systems, while only one DEG each was enriched in cellular processes, environmental information processing, and genetic information processing, respectively.

### Pfam domain analysis of DEGs

**Fig. 5:**
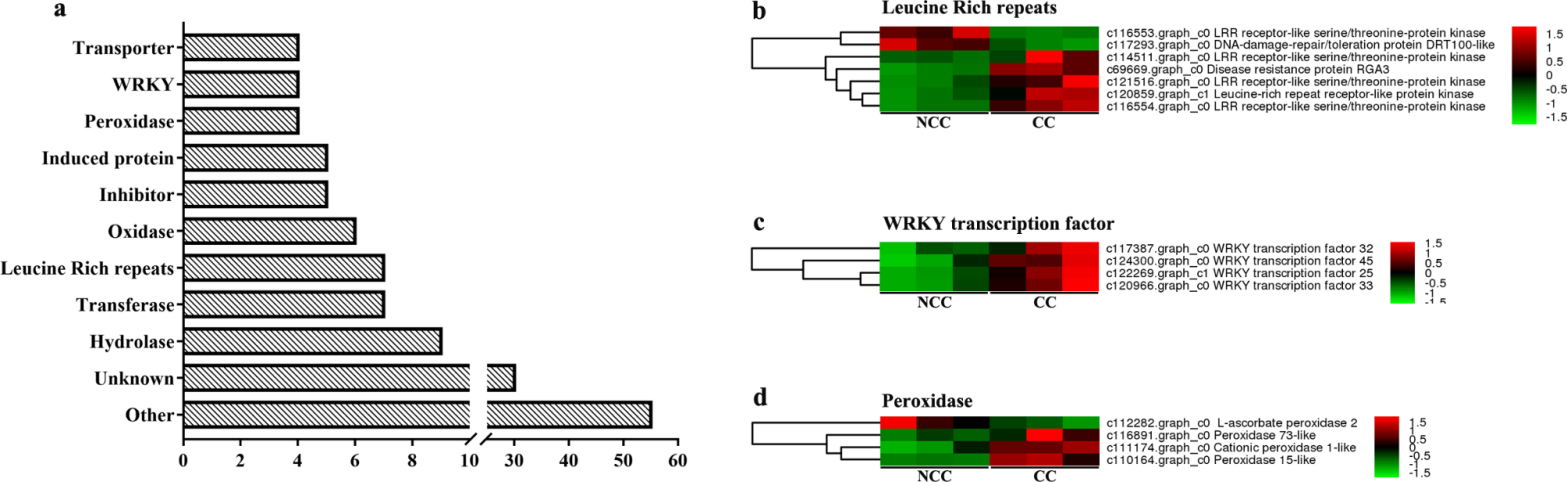
Pfam domain analysis of DEGs. (a) Pfam domain statistics. (b-d) Heatmap analysis of genes containing leucine-rich repeat, WRKY, and peroxidase domains, respectively

The Pfam protein domains of the DEGs were analyzed to predict their functions (Fig.5a). In this study, several Pfam domains, such as hydrolase, transferase, leucine-rich repeats, oxidase, inhibitor, induced protein, peroxidase, WRKY, and transporter, were particularly abundant among the DEGs. Previous studies have indicated that genes containing leucine-rich repeats, WRKY, and peroxidase domains play vital roles in plant tolerance mechanisms in response to various stresses [16–18]. Our results demonstrated that most of these three types of genes were up-regulated after CC. A total of seven leucine-rich repeats were found among the DEGs, with five up-regulated in response to CC (Fig.5b). Four up-regulated genes contained WRKY domains, and three of the four genes containing peroxidase domains were up-regulated in response to CC (Fig.5c-d).

### Real-time quantitative PCR (RT-qPCR) analysis

**Fig. 6:**
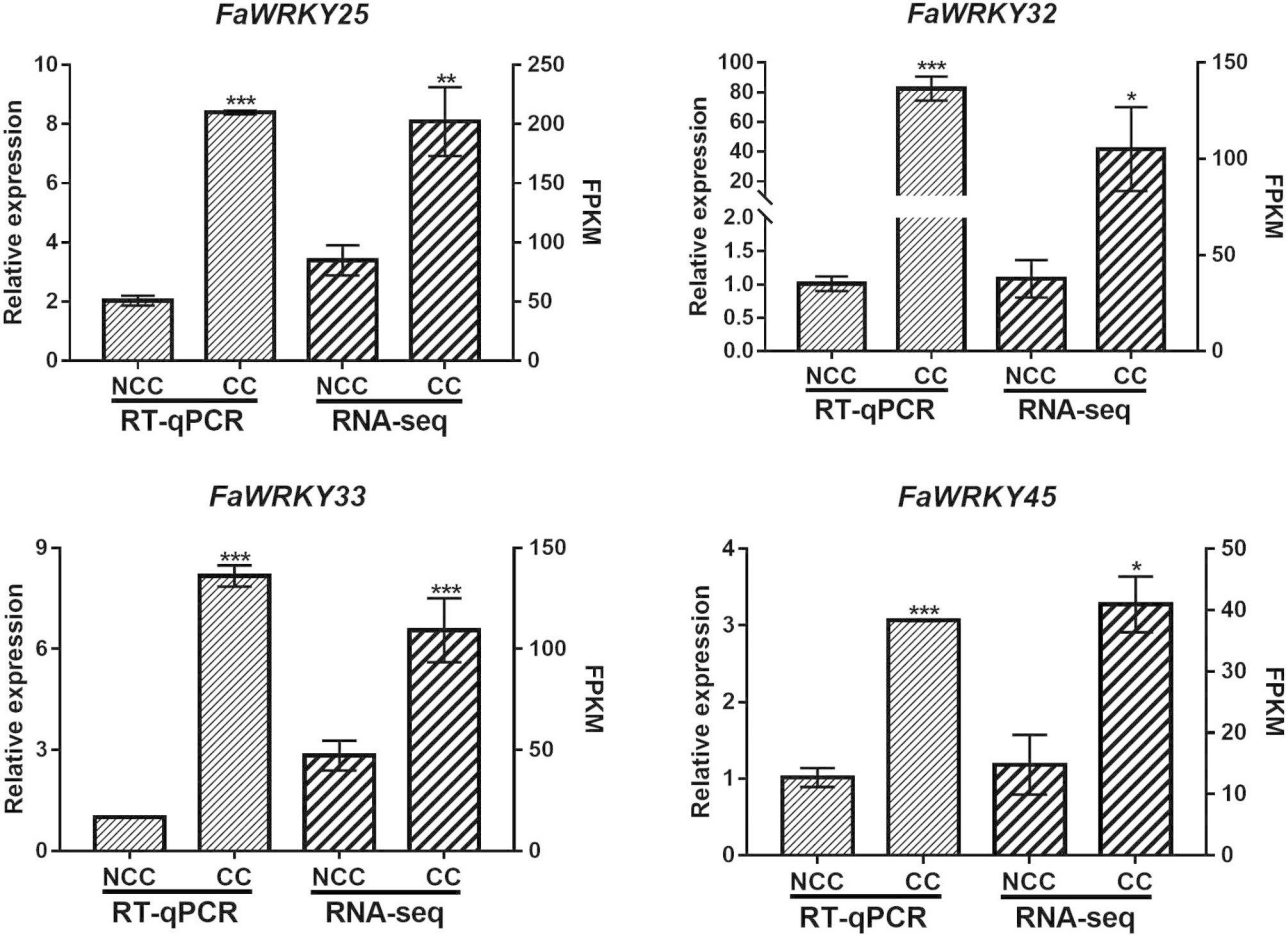
Expression profiles of four WRKY genes in strawberry roots under CC and NCC treatments. The bars indicate the standard deviation. The data were normalized to a DNA-binding protein (EU727547) gene using the 2^−ΔΔCT^ method. * *P* < 0.05, ** *P* < 0.01, and *** *P* < 0.001

Four WRKY family genes, comprising *WRKY25*(*c122269.graph_c1*), *WRKY32* (*c117387.graph_c0*), *WRKY33* (*c120966.graph_c0*) and *WRKY45* (*c124300.graph_c0*), were analyzed by RT-qPCR to verify the RNA-Seq results (Fig.6). The findings showed that all four of the WRKY genes were significantly up-regulated in the roots of plants subjected to CC. Although the fold changes of these four genes between NCC and CC were not always the same in the RT-qPCR and RNA-Seq results, the overall trend was consistent. The RT-qPCR data verified the accuracy of the RNA-Seq results, indicating that the RNA-Seq data were reliable.

## Discussion

Continuous cropping leads to changes in soil ecology, and these changes result in severe retardation of growth and even death in plants [9, 15]. Our results showed that CC led to significant decrease in strawberry plant biomass (Fig.1). Plants exposed to CC stress exhibit a characteristic set of stress response and adaptation strategies, including physiological, cellular and molecular changes. The results presented here focused on the molecular response of strawberry roots to CC stress. Our results provide important information on the potential molecular changes that occur following CC in strawberry roots.

Plants have evolved a series of defense strategies to cope with changeable environments [19]. To explore changes in gene expression in strawberry roots, we carried out genome-wide transcriptomic analyses of defensive responses to CC. In this study, we found that CC strongly influenced the fresh weights of the shoots and roots (Fig.1). This inhibition was accompanied by changes in gene expression [20]. Our results revealed a total of 136 DEGs following CC (Fig.2), including 49 up-regulated and 87 down-regulated genes. Changes in the expression of these genes may help to explain the problems caused by CC in strawberry.

In adapting to diverse and complex environments, plants have evolved a series of growth-defense tradeoffs to optimize fitness in response to resource limitations [21]. Previous studies have indicated that CC is a comprehensive stress, and the influence of consecutive CC practices on the transcriptome gene expression pattern is similar to the profile seen under salt and drought stresses in *Rehamannia glutinosa* [22]. Li et al. also found that *A. paniculata* could alter the expression of enzyme-encoding genes to better tolerate the stress of CC [14]. In this study, GO term and KEGG pathway enrichment analyses were performed to further understand the functions of the identified DEGs [23, 24]. The GO term enrichment results showed that many up-regulated DEGs were associated with defense-related GO terms and the most down-regulated DEGs were assigned to nutrient-related GO terms (Fig.3). These changes in gene expression in strawberry roots indicated a transition from growth to defense in response to CC. Long-term CC significantly disturbs the soil environment, which leads to changes in plant growth-defense tradeoffs [9]. When CC soil conditions are improved by soil amendment, plants increase their growth and reduce their defensive responses [15]. In contrast, when soil remains degraded after CC, plants do not grow well and instead reallocate their resources to defense responses. In this study, the KEGG pathway enrichment analysis showed that the most highly enriched pathways were phenylalanine metabolism, starch and sucrose metabolism, phenylpropanoid biosynthesis, glutathione metabolism, and plant-pathogen interaction, which are mainly defense-related pathways (Fig.4).

Phenylalanine is a substrate for both primary and secondary metabolic pathways and is necessary for cell survival [25]. In nature, phenylalanine is also a substrate for phenylpropanoid metabolism that generates a series of secondary metabolites [25, 26]. Phenylpropanoids play roles in all aspects of plant responses towards external stimuli [26]. They are the vital mediators of plant resistance to pests, and phenylpropanoid-based polymers, such as lignin, contribute substantially to plant tolerance towards environmental damage [26, 27]. Glutathione is an important component of the plant defense system, and it supports plants in coping with external stress by chelating or sequestering metals [28]. Plant resistance to pathogens requires the activation of plant-pathogen interaction pathways, the main one being the hypersensitive response (HR) [29]. The production of reactive oxygen species (ROS) is an important event in HR, and glutathione is involved in minimizing ROS-mediated cellular damage [28–30].

**Fig. 7:**
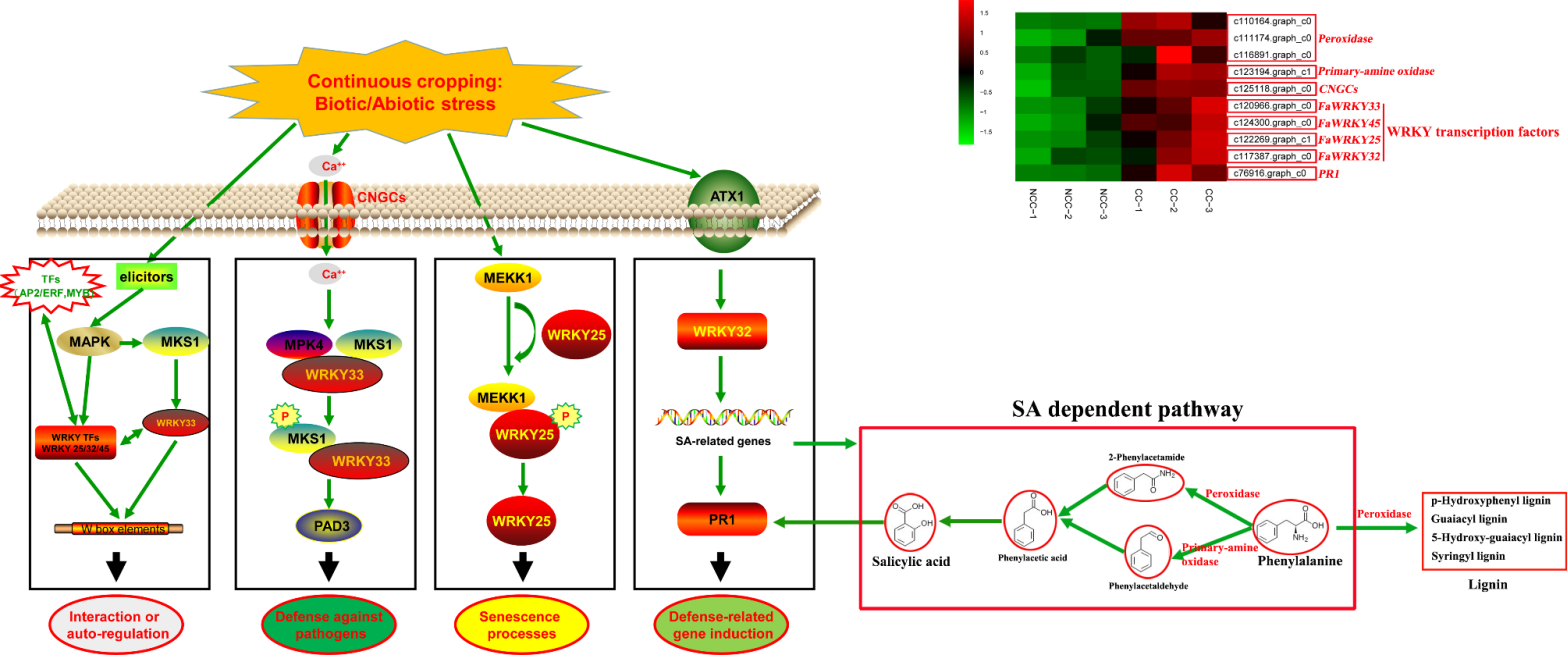
A possible gene expression regulatory network in response to CC. This figure was created using ScienceSlides 2005

During the process of plant evolution, complex defense mechanisms have evolved to cope with multiple environmental stresses. Several up-regulated genes identified in this study in response to CC, including WRKY transcription factors, peroxidase genes, the family of Cyclic Nucleotide-Gated Channels (CNGCs) and *Pathogenesis-related protein 1* (*PR1*), might play important roles during the response to CC in strawberry. According to the results of our transcriptome analysis, we mapped the gene expression regulatory network in response to CC (Fig.7). Our previous studies have shown that several WRKY gene members play important roles in CC in cultivated strawberry (*Fragaria* × *ananassa* Duch.) [31]. In this study, the biotic and abiotic stresses caused by CC acted as elicitors, leading to the up-regulation of *FaWRKY25/32/33/45*. Autoregulation and cross-regulation occur in *FaWRKY25/32/33/45* transcription factors through binding to the W-box [32]. Moreover, CNGCs play a critical role in responses to stressors, such as salt, heavy metals, drought, cold or heat, and pathogens [33]. In this study, *CNGC* family genes were significantly up-regulated in response to CC. A downstream gene, *FaWRKY33*, which responds to both biotic and abiotic stresses was also significantly up-regulated [34]. *FaWRKY25*, a homologous gene of *AtWRKY53*, participates in in the regulation of leaf senescence [35]. Therefore, the up-regulation of *FaWRKY25* might be one of the reasons for seedling death in response to CC. *FaWRKY32* is the homologous gene of *AtWRKY70*, and the overexpression of *AtWRKY70* leads to the constitutive expression of salicylic acid (SA)-induced genes [36]. In this paper, the expression of SA-related pathway genes, such as *peroxidase* and *primary-amine oxidase* genes, was significantly up-regulated under CC. In addition, SA and *FaWRKY32* can co-activate pathogenesis-related (PR) proteins. These proteins represent one of the several lines of defense found in plants against invading pathogens [37]. The PR proteins from some groups have been proven to have antimicrobial activity [38]. Under CC conditions, soil-borne plant pathogens in the rhizosphere accumulate in large quantities [9, 39, 40]. The latest data concerning plant pathogens shows that CNGCs and PR proteins are up-regulated after infection, which is consistent with our conclusions [37, 41]. Moreover, the functions of peroxidases in plants have been studied; these functions include the removal of H_2_O_2_, biosynthesis and degradation of lignin, and defensive responses to pathogen or insect attack [42–44]. The genes discussed above all play relevant roles in response to CC in strawberry.

Our previous studies have found that the problems associated with CC of strawberry crops are caused by dynamic changes in the soil environment, and these changes cause stress, affecting growth [9, 15]. The results of this study suggest that CC stress leads to downregulated expression of some nutrient-related genes. Therefore, roots reallocate more resources to enact potential changes, such as the upregulation of some defense-related genes, including WRKY transcription factors and *peroxidase* genes.

## Conclusion

We have identified genes that respond to CC stress using a transcriptomic analysis. Significant differences in gene expression were identified in the roots of strawberries subjected to CC and NCC. A group of DEGs, which may respond to CC, were identified, including some defense-related and nutrient-related genes. The upregulation of *CNGCs*, *WRKY* transcription factors, *PR1*, and *peroxidase* genes, which are mainly related to plant defense processes, suggest additional defensive strategies after CC stress. The conclusions from this study contribute to a deeper understanding of the defensive strategies of strawberry roots following exposure to CC.

### Methods

#### Plant materials and treatments

A cultivated strawberry variety, *Fragaria × ananassa* Duch. “Benihoppe”, was used in this experiment. The strawberry plants were provided by Beijing Academy of Forestry and Pomology Sciences, Haidian District, Beijing, China. All plants were cultivated in greenhouses under a temperature range of 10.4–26.7°C. The strawberry materials consisted of two groups: NCC and CC. The NCC strawberry plants were cultivated in an NCC greenhouse, planted with strawberries for the first time. The CC strawberry plants were cultivated in a CC greenhouse, which had been annually mono-cultivated with strawberry plants for more than 12 years. Each greenhouse contained 80 beds that were 100cm × 40cm × 40cm in size. The strawberry seedlings were cultivated in two rows per bed. The greenhouses were maintained under the same tillage management patterns, which included the following basic fertilizer applications: 29,985kg/ha farm manure with > 25% organic matter and 300kg/ha NPK fertilizer with ≥ 45% N + P_2_O_4_ + K_2_O.

The strawberry plant samples were collected randomly at harvest stage, and the roots were rinsed with water. The fresh weight was measured immediately, and the root samples were used for further RNA-Seq analysis. Each sample group contained three biological replicates (NCC-1, NCC-2, and NCC-3; CC-1, CC-2, and CC-3), and each biological replication included the roots of three seedlings [45]. All samples were snap-frozen in liquid nitrogen and kept at − 80°C.

### RNA extraction and cDNA library preparation and sequencing

RNA extraction of root samples taken from near the tip was performed as described previously [15]. The cDNA library preparation and sequencing of strawberry samples were carried out as described previously for RNA-Seq analysis [15]. The library preparations were also sequenced on an Illumina platform at Beijing BioMarker Technologies, Beijing, China. A total of six sets of raw reads were obtained for RNA-Seq analysis, including NCC-1, NCC-2, NCC-3; CC-1, CC-2, and CC-3.

### Data analysis

Transcriptome data processing was performed as described previously [15]. Moreover, the expression level was calculated using the reads mapped per 1000bp per million sequenced reads method (FPKM) [46]. A differential expression analysis of the two groups was performed using the DESeq R package (1.10.1). The Audic and Claverie method was employed to identify the DEGs between the NCC and CC groups [47]. The *P*-values were adjusted using Benjamini and Hochberg’s approach to control for the false discovery rate (FDR). Genes with a *P*-value < 0.01 and a |fold change| ≥ 2 according to DESeq were considered to be significant DEGs. All significant DEGs were mapped to the GO and KEGG databases using the GOseq R package and KOBAS software, respectively [23, 48]. The statistical analysis was performed using an independent-samples *t* test with SPSS 20.0 (SPSS Inc., USA).

### RT-qPCR analysis

In this study, four WRKY DEGs were chosen, and the primers are listed in Table S1. The primers were obtained from qPrimerDB (https://biodb.swu.edu.cn/qprimerdb/) [49]. The RT-qPCR was performed as previously described [15]. Gene relative expression was normalized relatively to the strawberry housekeeping gene DNA-binding protein EU727547 using the 2^−ΔΔCT^ method [50–52]. Each reaction was repeated using three independent biological and technical replicates.

## Electronic supplementary material

Below is the link to the electronic supplementary material.

Supplementary Material 1

## Data Availability

The RNA-Seq datasets used in the current study are available in the NCBI Sequence Read Archive Project - PRJNA869951 (https://www.ncbi.nlm.nih.gov/bioproject/PRJNA869951). All other data generated or analyzed during this study are included in this published article and its supplementary information files.

## Abbreviations

NCC: Noncontinuous cropping
CC: continuous cropping
DEGs: differentially expressed genes
cDNA: complementary DNA
FDR: false discovery rate
FC: fold change
GO: Gene Ontology
KEGG: Kyoto Encyclopedia of Genes and Genomes
CNGCs: Cyclic Nucleotide-Gated Channels
PR1: Pathogensis-Related protein 1
