# Supplementary material for: Transcriptomic analysis provides insight into defensive strategies in response to continuous cropping in strawberry (Fragaria × ananassa Duch.) plants
Source: BMC Plant Biol. 2022 Oct 7;22:476. doi: 10.1186/s12870-022-03857-6 (PMC9540695; doi:10.1186/s12870-022-03857-6)
Supplement: Supplementary file 1 — Supplementary Material 1 [file 12870_2022_3857_MOESM1_ESM.docx]

Table S1 The primers of RT-qPCR.

| Gene |  | Sequences 5'→3' | Annealing Temperature |
| --- | --- | --- | --- |
| FaWRKY25 | Forward | CCTTTAGGAACACACAAAGCTG | 58℃ |
|  | Reverse | TCTTCCAAATTCTCAGTGTCGA |  |
| FaWRKY32 | Forward | GTACCGAAACGTTTAAGGATCG | 58℃ |
|  | Reverse | AGAATCGTCTTTTGTCCGTACT |  |
| FaWRKY33 | Forward | CTCACAAGCAACATGAACATGA | 58℃ |
|  | Reverse | TGTTCTTGTTGCTCAGTTGAAC |  |
| FaWRKY45 | Forward | AGGACATTCTTGGAGCCAAATA | 58℃ |
|  | Reverse | TGCCTTCCTCGGTAAGTAATTT |  |
